# Anti-allergic Inflammatory Effects of the Essential Oil From Fruits of *Zanthoxylum coreanum Nakai*

**DOI:** 10.3389/fphar.2018.01441

**Published:** 2018-12-12

**Authors:** Rui Hong Guo, Jung Up Park, Se Jin Jo, Jae Hun Ahn, Jong Hwan Park, Ji Yoon Yang, Sung Suk Lee, Mi Jin Park, Young Ran Kim

**Affiliations:** ^1^College of Pharmacy and Research Institute of Drug Development, Chonnam National University, Gwangju, South Korea; ^2^Laboratory Animal Medicine, College of Veterinary Medicine, Chonnam National University, Gwangju, South Korea; ^3^Division of Wood Chemistry, Department of Forest Products, National Institute of Forest Science, Seoul, South Korea

**Keywords:** *Zanthoxylum coreanum Nakai*, degranulation, inflammatory mediators, iNOS, COX-2, NF-κB, MAPK, atopic dermatitis

## Abstract

*Zanthoxylum coreanum Nakai* is a rare shrub which grows in Korea and China. Pericarp of *Z. coreanum* has been used as a crude medicine, but there are few researches about the pharmacologic activities. The present study was designed to investigate the anti-allergic inflammatory activities of the essential oil from fruits of *Zanthoxylum coreanum Nakai* (*ZCO*). Our findings showed that *ZCO* inhibited both the IgE-antigen complex or PMA/A23187-induced β-hexosaminidase release and IL-4 production dose-dependently in RBL-2H3 mast cells, and confirmed that *ZCO* at the tested concentrations did not show cytotoxicity to RBL-2H3 cells by MTS assay. Additionally, we found that *ZCO* showed the significant inhibition on LPS-induced overproduction of TNF-α, IL-6 and NO. Consistently, the protein levels of iNOS and COX-2 were also remarkably decreased by *ZCO* treatment. Herein, Our mechanistic studies revealed that *ZCO* significantly suppressed the activation of transcription factor NF-κB in PMA-activated 293T cells, and further inhibited NF-κB p65 translocation into the nucleus in LPS-stimulated RAW264.7 cells. Further investigation identified that *ZCO* down-regulated LPS-induced phosphorylation of MAPK (JNK, ERK, and p38) signal pathway. For incremental research, we established an DNCB-induced atopic dermatitis model in BALB/c mice, and found that *ZCO* remarkably inhibited DNCB-induced ear swelling and AD-like symptoms. Based on these findings, *ZCO* is suggested to have a therapeutic potential for the allergic inflammatory diseases.

## Introduction

Allergic diseases are inflammatory disorders which have become a worldwide clinical health problem. With the allergy patients increasing annually due to various factors, approximately 10 ∼ 20% of the world population is affected by allergies (Ring et al., 2014; Do et al., 2017; Appel et al., 2018). Most allergy patients are genetically predisposed to produce IgE antibody. Mast cells express a high-affinity IgE receptor on membranes, which is important to the pro-inflammatory allergic response. When an IgE-antigen binds with FcεRI (high-affinity receptor for the Fc region of immunoglobulin E), the receptor is activated, and occurs complex biological reactions causing allergic reactions, including inflammatory disorder (Bochner and Schleimer, 2001; Yang et al., 2016; Appel et al., 2018). Also, a combined treatment with PMA/A23187 has been used widely for mast cell activation, because they are known to induce the generation of inflammatory cytokines (Hong et al., 2010; Vo et al., 2011). RBL-2H3 cells, a mast cell line, are originated from rat basophilic leukemia, and have been used to study IgE-FcεRI interactions and degranulation. Furthermore, RBL-2H3 cells are a useful model for *in vitro* screening of anti-allergic agent candidates (Tang et al., 2015). β-hexosaminidase, a granule-associated exoglycosidase, is stored in secretory granules of mast cells, and has been used to monitor mast cell degranulation just as histamine has been used (Dinneswara Reddy et al., 2012; Zhang et al., 2013). During the pathogenesis of allergic disease, IL-4 is also crucial for the induction of IgE synthesis and mast cell development (Nabeshima et al., 2005).

Inflammation is a complex mechanism involving the activation and deactivation of immune cells, which could result in cellular and tissue damage causing the chromic disease (Alessandri et al., 2013; Ahmad et al., 2017). Macrophages are key immune cells in initiating and maintaining the inflammatory response. Lipopolysaccharide (LPS), a principle component of the outer membrane of Gram-negative bacteria, could induce the macrophage cells’ inflammation reaction, and stimulate the production of inflammatory mediators such as nitric oxide (NO), tumor necrosis factor (TNF)-α and IL-6 (Yi et al., 2013; Chen et al., 2014). This could be used to assess the anti-inflammatory activities of samples. Nuclear factor kappa B (NF-κB) is an important regulator of allergic inflammation, which controls the expression of pro-inflammatory mediators (Kim et al., 2018). When stimulated, NF-κB translocates to the nucleus, and regulates the expression of various transcription factors leading to the expression of pro-inflammatory enzymes like cyclooxygenase 2 (COX-2) and inducible nitric oxide synthase (iNOS), which are responsible for the stimulation of inflammatory signaling molecules (Ahmad et al., 2017). Apart from NF-κB, MAPKs also plays an important role in the regulation of the cell growth, cell differentiation, and cellular pro-inflammatory molecules (Huang et al., 2012; Song et al., 2018).

Essential oils have been used to treat allergy and inflammation as cosmetic materials. In the present study, we obtained essential oils by hydro-distillation from some tree plants or fruits and screened the anti-allergic or inflammatory activities. The essential oil from fruits of *Zanthoxylum coreanum Nakai* (*ZCO*) showed the most inhibitory activity on the mast cell degranulation. *Zanthoxylum coreanum Nakai*, Korean lime tree, is a rare shrub which only grows in Korea and China. In Korea, it is called wang-cho-pi tree. Pericarp of *Z. coreanum* has been used as a crude medicine for the treatment of ozena, rheumatoid, nasal sinusitis, sore throat, etc. in Korea. Previous study has reported that *Z. coreanum* showed the antiviral activity against picornaviruses (Choi, 2016). However, there are few researches about the pharmacologic activities of *Z. coreanum.* Here, we studied the anti-allergic inflammatory effects of *ZCO* on degranulation and IL-4 production in RBL-2H3 cells, inflammatory cytokines production such as NO, TNF-α, and IL-6, and the protein expression levels of iNOS and COX-2 in LPS-induced RAW264.7 macrophage cells. For incremental research, we investigated the mechanism related to the NF-κB and MAPKs signal pathways. In addition, *in vivo* experiment was designed to investigate the effect of *ZCO* on DNCB-induced AD-like symptoms. To our knowledge, this is the first evidence for the effects of *ZCO* on allergic inflammation.

## Materials and Methods

### Plant Material

The fruits of *Z. coreanum* were used in this study. The fruits were collected in experimental forest of National institute of forest science located on Jin-ju city, republic of Korea, in August 2017. Taxonomical identifications were established by the ecologist Dr. Hwa-Ja Hyeon at warm temperate and subtropical forest research center of national institute of forest science and the voucher specimen code was WTFRC 10030535.

### Essential Oil Extraction

The fruits of *Z. coreanum* were hydro-distillated at atmospheric pressure, using a clevenger-type apparatus. A 10 L round-bottom flask containing 2099.8 g of fruits was placed in heating mantle. In this flask, the fruits were mixed in 5 L distilled water. Then the flask was connected with clevenger type apparatus. The fruits of *Z. coreanum* were extracted for 14 h. The collected essential oil was dried over anhydrous sodium sulfate and filtered through 0.45 μm membrane disk-filter. The essential oil obtained was transferred to sealed dark vials and stored at 4°C for further analysis.

The essential oil content was determined on the basis of oven dry matter and measurements were carried out in triplicates. The yield (%) of essential oil was calculated using the following formula:

$$Essential\;oil\;yield(\%)\;=\;\frac{\text{mass}\;\text{of}\;\text{essential}\;\text{oil}\;\text{obtained}\;\text{(g)}}{\text{mass}\;\text{of}\;\text{oven}\;\text{dry}\;\text{matter}\;\text{(g)}}\times 100$$

### Gas Chromatography–Mass Spectrometry (GC–MS) Analysis

Coupled GC-MS analysis was performed on a Thermo Scientific Model ISQ LT equipped with both a flame ionization detector (FID) and a mass spectrometer (MS). GC/MS analysis solution was prepared by dissolving 4 μL oil to 1.0 mL dichloromethane solution (containing 100 ppm methyl undecanoate), and 1 μL was injected. A VF-5MS GC column (60 m × 0.25 mm × 0.25 μm film thickness; Agilent Technologies) was used. Carrier gas was helium at a constant flow rate of 1.0 mL/min. Injection temperature was 250°C with a split ration 1:20. The oven temperature was maintained at 50°C for 5 min, then increased at 10°C/min to 65°C held for 30 min, then at 5°C/min to 120°C held for 10 min, then at 5°C/min to 210°C held for 10 min, and finally at 20°C/min to 325°C held for 10 min. For FID detection, temperature was set to 300°C, air flow to 350.0 mL/min, hydrogen flow to 35.0 mL/min, and make-up gas (helium) flow to 40.0 mL/min. Mass interface temperature was 250°C and ion source temperature was 250°C. Mass scan data was acquired in EI mode at 0.2 s scan time rate with a scan range from 35 amu to 550 amu.

The identification of peaks was performed by comparing peak average mass spectrum of peak with an electronic library database (NIST/EPA/NIH Mass Spectral Library, version 2.0 g). In addition, the identity of the compounds was assigned by comparison of the koviats retention indices (KI, Table 1) determined in relation to a homologous series of n-alkanes (C7-C30).

**Table 1:** Chemical compositions of *ZCO*.

RT	Constituent	Area %	KI^a^	Identifi
				cation^b^
19.36	α -Thujene	0.59	917.77	MS, KI
20.17	(-)-*α*-Pinene	16.56	923.62	MS, KI
22.22	Camphene	0.06	938.43	MS, KI
25.24	Sabinene	10.81	960.24	MS, KI
26.01	β-Pinene	0.30	965.80	MS, KI
27.98	β-Myrcene	0.32	980.03	MS, KI
34.76	*o*-Cymene	3.56	1029.00	MS, KI
35.62	Limonene	2.63	1035.21	MS, KI
35.91	*β*-Phellandrene	3.15	1037.31	MS, KI
37.39	*β*-Ocimene	24.48	1048.00	MS, KI
39.15	α -Ocimene	2.40	1060.71	MS, KI
40.82	γ-Terpinene	0.17	1072.77	MS, KI
42.21	Sabinene hydrate	0.07	1082.81	MS, KI
42.62	α-Pinene oxide	0.39	1085.77	MS, KI
43.56	Terpinolene	0.07	1092.56	MS, KI
44.27	3-Methyl-2-(3-methylbut-2-enyl)-furan	0.15	1097.69	MS, KI
44.80	Linalool	10.09	1103.31	MS, KI
45.95	Camphor	0.07	1121.42	MS, KI
46.52	cis-ρ-Menth-2-en-1-ol	0.48	1130.39	MS, KI
46.70	(4E,6Z)-Alloocimene	0.69	1133.23	MS, KI
47.52	β-Pinene oxide	0.18	1146.14	MS, KI
47.69	p-Menth-3-en-1-ol	0.46	1148.82	MS, KI
50.04	4-Carvomenthenol	11.61	1185.83	MS, KI
50.65	4-Isopropyl-2-cyclohexenone	0.64	1195.43	MS, KI
51.07	α -Terpineol	1.74	1201.69	MS, KI
52.09	p-Pent-1-en-3-ol,cis-(+)-	0.26	1217.95	MS, KI
53.14	Ascaridole	0.13	1228.61	MS, KI
57.77	Bornyl acetate	0.05	1288.82	MS, KI
58.70	Limonene dioxide	0.02	1301.19	MS, KI
64.33	β-Elemene	0.10	1396.61	MS, KI
67.00	Humulene	0.02	1461.44	MS, KI
67.51	α-Farnesene	0.20	1461.44	MS, KI
68.18	α-Muurolene	0.03	1490.80	MS, KI
69.55	(+)-δ-Cadinene	0.19	1531.75	MS, KI
71.79	β-Caryophyllene oxide	0.45	1603.38	MS, KI
73.48	τ-Muurolol	0.18	1666.92	MS, KI
73.80	(-)-α-Cadinol	0.49	1678.95	MS, KI

^a^*Kovats index on a DB-5 column in reference to n-alkanes. ^*b*^MS, NIST/EPA/NIH Mass Spectral Library; KI, Kovats index*.

### Cell Culture and Reagents

The rat basophilic leukemia cell line RBL-2H3 was supplied from the Korean Cell Line Bank (Seoul, South Korea). RBL-2H3 cells were cultured in Minimum Essential Medium (MEM) (Welgene, South Korea) supplemented with 10% FBS (Gibco, Rockville, MD, United States) and 1% penicillin-streptomycin under an atmosphere of 5% CO_2_ in a humidified 37°C incubator. RAW264.7 macrophage cells, IgEL b4 cells and 293 T cells were purchased from American Type Culture Collection (Manassas, VA, United States) and were cultured in Dulbecco Modified Eagle’s medium (DMEM) with 10% FBS and 1% penicillin-streptomycin. Calcium ionophore A23187 and phorbol 12-myristate 13-acetate (PMA) were purchased from Sigma-Aldrich (St. Louis, MO, United States). Quercetin, 1-Chloro-2,4-dinitrobenzene (DNCB) and dexamethasone were purchased from Sigma-Aldrich (St. Louis, MO, United States). Albumin from bovine serum, 2,4-dinitrophenylated (DNP-BSA) was purchased from Invitrogen by Thermo Fisher Scientific (Eugene, OR, United States). rIL-4 and mTNF-α were purchased from R&D system Inc (Minneapolis, MN, United States). mIL-6 was purchased from Biolegend (San Diego, CA, United States).

Stock solutions of *ZCO* were diluted using the mixture solution of DMSO and ethanol (1:1) to a concentration of 20% and filter-sterilized, and then diluted with phosphate buffered saline (PBS) for the working concentration.

### Measurement of Cell Viability

RBL-2H3 cells, RAW264.7 cells, and 293T cells were seeded into 96-well plates (SPL life Sciences Co., Pocheon, South Korea) at 1.5 × 10^5^ cells/mL overnight under an atmosphere of 5% CO_2_ in a humidified 37°C incubator, then the cells were treated with *ZCO* at the concentration of 0.0025, 0.005, 0.01% for 24 h. Cell proliferation was assayed using 3-(4,5-dimethylthiazol-2-yl)-5-(3-carboxymethoxyphenyl)-2-(4-sulfophenyl)-2H-tetrazolium (MTS) (Promega, Madison, WI, United States) according to manufacturer’s instructions, and absorbance was read with a microplate reader (BioTek, Winooski, VT, United States) at 490 nm.

### β-Hexosaminidase Release by RBL-2H3 Cells

β-hexosaminidase is a marker of mast cell degranulation (Dinneswara Reddy et al., 2012). RBL-2H3 cells were incubated into 48-well plate (2 × 10^5^ cells/mL) in MEM with 10 % FBS and IgE anti-DNP at 37°C overnight. After washing 2 times with siraganian buffer (SB) pH 7.2, 119 mM NaCl, 5 mM KCl, 0.4 mM MgCl_2_^⋅^6H_2_O, 25 mM PIPES [piperazine-N, N′-bis (2-ethanesulfonic acid), 40 mM NaOH], the cells were incubated with SBC buffer (siraganian buffer containing 5.6 mM glucose, 1 mM CaCl_2_, and 0.1% BSA). Then cells were pretreated with *ZCO* at the concentration of 0.0025, 0.005, 0.01% or the positive control quercetin (20 μM) respectively, for 1 h prior to stimulation with DNP-BSA (1 μg/mL) (Thermo Fisher Science, OR, United States) for 30 min at 37°C and stopped on ice for 5 min, and the cell supernatants (50 μL) collected by centrifugation were incubated with 50 μL of 1 mM 4-nitrophenyl-N-acetyl-β-D-glucosaminide (Sigma, St Louis, MO, United States) in 0.1 M sodium citrate (pH 4.5) at 37°C for 1 h. The reaction was terminated with 200 μl/well of carbonate buffer containing 0.1 M Na_2_CO_3_ and 0.1 M NaHCO_3_ (pH 10), and the absorbance was measured at 405 nm with a microplate reader (BioTek, Winooski, VT, United States).

In addition, RBL-2H3 cells incubated into 48-well plate (3 × 10^5^ cells/mL) in MEM with 10% FBS overnight were washed 2 times with SB buffer, and then incubated with SBC buffer. Cells were then pretreated with *ZCO* at the concentration of 0.0025, 0.005, and 0.01% or quercetin (20 μM) for 1 h prior to stimulation with 30 ng/mL of PMA and 350 ng/mL of A23187 for 1 h at 37°C, and stopped on ice for 5 min, then cell supernatants were collected for β-hexosaminidase activity assay, as described previously (Su et al., 2018).

### Rat IL-4 Secretion by RBL-2H3 Cells

To measure the IL-4 concentrations in the culture media, RBL-2H3 cells were incubated into 48-well plate (2 × 10^5^ cells/mL) in MEM with 10% FBS and IgE anti-DNP at 37°C overnight. After washing 2 times with MEM, the cells were pretreated with *ZCO* at the concentration of 0.0025, 0.005, and 0.01% or quercetin (20 μM) respectively, for 2 h and then treated with DNP-BSA (1 μg/mL) for 16 h at 37°C. The cell-free supernatants were collected and IL-4 concentrations were measured using ELISA kit (R&D, Minneapolis, MN, United States) according to the manufacturer’s instruction.

In addition, RBL-2H3 cells were incubated into 48-well plate in MEM with 10% FBS overnight and pretreated with *ZCO* at the concentration of 0.0025, 0.005, and 0.01% or quercetin (20 μM) respectively, for 2 h. Then cells were stimulated with 30 ng/mL of PMA and 350 ng/mL of A23187 for 16 h. The supernatants were collected for the measurement of IL-4 levels.

### NF-κB Reporter Assay

293T cells were seeded into poly-D-lysine hydrobromide (Sigma, MO, United States) coated 96 well plate at 1.5 × 10^5^ cells/mL, and then transient transfection of a reporter plasmid pNF-κB-SEAP (Clontech Laboratories, Palo Alto, CA, United States) was performed to cells with HilyMax for 4 h. After transfection, the cell medium was replaced with fresh DMEM overnight. Then cells were treated with *ZCO* at the concentration of 0.0025, 0.005, and 0.01% or the positive control Bay11-7082 (20 μM) for 2 h prior to stimulation with 3 ng/mL of PMA (Sigma, St. Louis, MO, United States) for 24 h. The supernatants (10 μL) were incubated with Quanti-Blue (100 μL) (Invitrogen, Carlsbad, MA, United States) for 1 h, and the absorbance was read at 630 nm with an ELISA microplate reader (BioTek, Winooski, VT, United States).

### NF-κB Staining

RAW264.7 cells were plated into an 8-well glass chamber plate (Thermo Fisher Scientific, United States) at 1 × 10^5^ cells/mL overnight. The cells were pretreated with *ZCO* (0.01%) or Bay11-7082 (20 μM) for 2 h prior to stimulation with 1 μg/mL of LPS (Sigma Co., MO, United States) for 2 h, and then fixed with 4% paraformaldehyde (Molecular Probes, Inc., Eugene, OR, United States) and permeabilized with 0.1% triton for 15 min. The NF-κB p65 protein was detected by immunostaining using a polyclonal-anti-NF-κB p65 antibody (Invitrogen, Carlsbad, MA, United States) and Alexa Fluor 488-conjugated anti-rabbit IgG antibody (Molecular Probes Invitrogen, MA, United States). Actin was visualized by staining with Alexa Fluor 594 conjugated phalloidin. The cells were mounted by using Prolong gold anti-fade reagent with DAPI (Molecular Probes). Bay 11-7082 (Sigma, MO, United States) was used as the positive control for the NF-κB inhibitors. Fluorescence images were acquired using Laser Scanning Confocal Microscope System (Leica TCS SP5/AOBS/Tandem, Germany) at Korea Basic Science Institute, Gwangju Center).

### Nitric Oxide Analysis

RAW264.7 cells were plated into 48-well plates (SPL Life Sciences Co., Pocheon, South Korea) at 2 × 10^5^ cells/mL and treated with *ZCO* (0.0025, 0.005, and 0.01%) or the positive control Bay11-7082 (20 μM) for 2 h before stimulation with 500 ng/mL of LPS. After 24 h of incubation, nitric oxide (NO) production in the culture supernatants was measured using Griess Reagent [1% sulfanilamide in 5% H_3_PO_4_, 0.1% N-(1-naphthyl)-ethylenediamine dihydrochloride] and incubated for 30 min at room temperature. The absorbance was read at 570 nm using an ELISA microplate reader (BioTek, Winooski, VT, United States).

### Measurement of Pro-inflammatory Cytokines Production

RAW264.7 cells were seeded into 48-well plates (SPL Life Sciences, Pocheon, South Korea) at 2 × 10^5^ cells/mL and treated with *ZCO* (0.0025, 0.005, and 0.01%) or the positive control Bay11-7082 (20 μM) for 2 h before stimulation with 500 ng/mL of LPS. The supernatants were collected after incubation for 24 h to measure the TNF-α (R&D system, Minneapolis, MN, United States) and IL-6 (Biolegend, CA, United States) with an ELISA kit according to manufacturer’s instructions.

### Western Blot Analysis

RAW264.7 cells cultured in a 6-well plates were harvested in an ice-cold cell culture lysis reagent (Promega, Madison, WI, United States). The protein of the cell lysates was determined using the Bradford reagent (Bio-Rad laboratories, CA, United States). Equal amounts of protein extracts (20 μg) for iNOS or COX-2 determination or 50 μg protein for MAPK pathway determination in a lysis buffer were subjected to 10% SDS-PAGE analysis and then electro-transferred onto polyvinylidene fluoride membranes (PVDF) (Millipore, Bedford, MA, United States). Following blocking of the non-specific binding sites with 5% skin milk at room temperature for 2 h, the membrane were incubated with primary antibodies directed against polyclonal antibodies specific to COX-2 (1:1000, Cell Signaling Tech., Danvers, MA, United States), iNOS (1:1000, Santa Cruz, CA, United States), or β-actin (1:1000, Santa Cruz, MA, United States), p-p38, p-38 (1:500, Cell signaling Tech, MA, United States), p-ERK, ERK (1:500, Cell signaling Tech, MA, United States), p-JNK, JNK (1:500, Cell signaling Tech, MA, United States), and NF-κB p65 (1:1000, Santa Cruz, MA, United States), IkB-α (1:1000, Cell signaling Tech, MA, United States), LaminB (1:1000, Santa Cruz, MA, United States) overnight at 4°C, and then were incubated with horseradish-peroxidase- conjugated second antibodies at room temperature for 1 h. Immunoreactive proteins were visualized using an ECL Western blot detection system (Advansta, Menlo Park, CA, United States).

### Subcellular Fractionation

RAW264.7 cells cultured in 6-well plates were pretreated with *ZCO* (0.005 and 0.01%) or Bay11-7082 (10 μM) for 4 h, and then stimulated with LPS (1 μg/mL) for 30 min. RAW264.7 cells were lysed in a hypotonic buffer (EDTA 5 mM, dithiothreitol (DTT) 5 mM, HEPES 10 mM (pH 7.5), MgCl_2_ 5 mM, protease inhibitor cocktail) on ice for 30 min, and then centrifuged at 1300 × *g* for 10 min at 4°C. The supernatants with cytosolic proteins were transferred into new iced tubes. The pellets were lysed with hypertonic buffer [HEPES 10 mM (pH 7.9), MgCl_2_ 1.5 mM, NaCl 100 mM, DTT 0.5 mM, EDTA 0.1 mM, protease inhibitor cocktail] on ice for 1 h, and then centrifuged at 13000 × *g* for 10 min. Supernatants with nuclear protein were collected.

### Induction of Atopic Dermatitis in the Mouse Ear

All animal experiments were approved by the Institutional Animal Care and Use Committee of Chonnam National University, and the protocol was approved by the Committee. The induction of AD-like lesions by DNCB was performed according to our previous research (Park et al., 2017). Female BALB/c white mice (7 weeks) were divided into 5 groups (*n* = 4), and control group was sensitized by the application of 150 μL of the mixture of acetone and olive oil (4:1), and other groups were sensitized by the application of 2 % 1-Chloro-2,4-dinitrobenzene (DNCB, Sigma, MO, United States) dissolved in the mixture of acetone and olive oil (4:1) on the shaved abdominal skin. After 4 days’ rest, 20 μL of *ZCO* (1 and 2%) or dexamethasone (1%, Sigma, MO, United States) was pretreated in both sides of the ears for 30 min, and followed by application of 20 μL of 1% DNCB. After 3 h, repeated one time. On day 6, the same application was repeated as the day 5. From day 7, ears were treated with 20 μL of *ZCO* or dexamethasone only. Ear thickness was daily measured with a digimatic micrometer (Ozaki seisakusho, Tokyo, Japan) (Approval Number: CNU IACUC-YB-2018-72).

### Histological Analysis

The ears of the mice were fixed in 10% neutral formalin for 24 h. The tissues were processed with alcohol and xylene series and embedded in paraffin. The paraffin blocks were sectioned at 2 μm, and then stained with hematoxylin and eosin, and examined under microscopy (Kang et al., 2017). Histological changes (acanthosis, thickening of the subepidermal layer, cellular infiltration) were evaluated semiquantitatively on a grade of 1–5 (1 = no change from negative control group; 5 = maximum change).

### Statistical Analysis

All statistical calculations were performed using GraphPad Prism version 5.01 (GraphPad Software, San Diego, CA, United States). All experiments were repeated at least three times, and the data were obtained at least in triplicate. The results are presented as the means ± SEM. Statistical comparisons were evaluated using student’s *t*-test with a *P*-value < 0.05 considered statistically significant.

## Results

### Chemical Composition of *ZCO*

The yield of *ZCO* was about 2.1%. The chemical compositions of *ZCO* are shown in Table 1 and 37 constituents were identified. β-Ocimene was predominant component, accounting for 24.48% of the total. It was followed by (-)-α-pinene (16.56%), 4-carvomenthenol (11.61%), sabinene (10.81%), linalool (10.09%), o-cymene (3.56%), β-phellandrene (3.15%), limonene (2.63%), and α-terpineol (1.74%) (Table 1).

### Effect of *ZCO* on the Cell Viability

**FIGURE 1:**
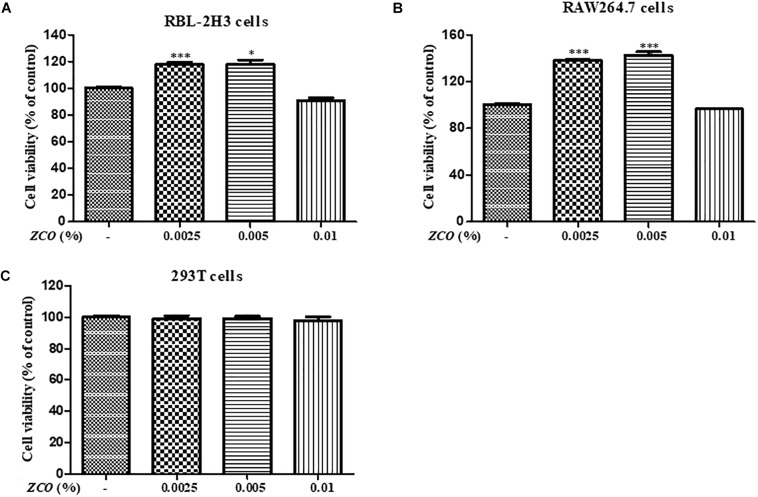
Effect of *ZCO* on the cell viability. **(A)** RBL-2H3 cells, **(B)** RAW264.7 cells, and **(C)** 293T cells were seeded into 96-well plate overnight, and then were treated with *ZCO* (0.0025, 0.005, and 0.01%) for 24 h. The cell viability was tested by MTS assays. The data are representative of three independent experiments and expressed as mean ± SEM (^∗^*P* < 0.05; ^∗∗∗^*P* < 0.001 versus with control group).

Effect of *ZCO* on cell viability was evaluated by MTS, respectively, in RBL-2H3, RAW264.7 and 293T cells to ensure that its anti-allergic inflammatory effects was not due to the cell death in each condition. Treatment with *ZCO* for 24 h at the concentrations of 0.0025, 0.005, and 0.01% showed no significant cytotoxic effects. In addition, *ZCO* at the concentrations of 0.0025 and 0.005% showed the increased cell growth of RBL-2H3 and RAW264.7 cells (Figure 1).

### Effect of *ZCO* on β-Hexosaminidase Secretion in RBL-2H3 Cells

**FIGURE 2:**
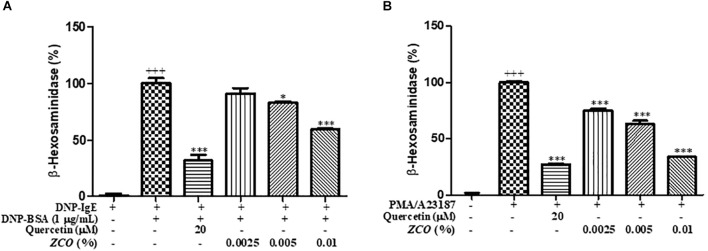
Effect of *ZCO* on β-hexosaminidase secretion in RBL-2H3 cells. **(A)** RBL-2H3 cells incubated into 48-well plate (2 × 10^5^ cells/mL) in MEM with 10% FBS and IgE anti-DNP a 37°C overnight were washed with SB buffer, and then were pretreated with *ZCO* (0.0025, 0.005, and 0.01%) or quercetin (20 μM) with SBC buffer for 1 h prior to stimulation with DNP-BSA (1 μg/mL) for 30 min. The supernatants were collected for β-hexosaminidase assay. Experiments were conducted in quadruplicate and expressed as mean ± SEM (^∗^*P* < 0.05; ^∗∗∗^*P* < 0.001 versus with DNP-BSA group). **(B)** RBL-2H3 cells incubated into 48-well plate overnight were washed with SB buffer, and then pretreated with *ZCO* or quercetin with SBC buffer for 1 h prior to stimulation with 30 ng/mL of PMA plus 350 ng/mL of A23187 for 1 h. The supernatants were collected for the detection of β-hexosaminidase (^∗∗∗^*P* < 0.001 versus with PMA/A23187 group). ^+++^means the significance compared with the mock cells (deal with nothing).

Mast cells contain a large amount of cytoplasmic granules, which activation caused the process of degranulation (Zeng et al., 2016). The release of β-hexosaminidase can be used to quantify the extent of degranulation (Huang et al., 2015). Pre-treatment with *ZCO* significantly suppressed the degranulation either in IgE-antigen complex or PMA/A23187-stimulated RBL-2H3 cells. In addition, *ZCO* treatment reduced the degranulation in RBL-2H3 cells in a dose-dependent manner, and *ZCO* at the concentration of 0.01% in the PMA/A23187- stimulated RBL-2H3 cells showed the similar inhibition of β-hexosaminidase release to the positive control quercetin, with an IC_50_ value of 0.0066%, which indicated that *ZCO* may be a promising new anti-allergic agent (Figure 2).

### Effect of *ZCO* on IL-4 Production in RBL-2H3 Cells

**FIGURE 3:**
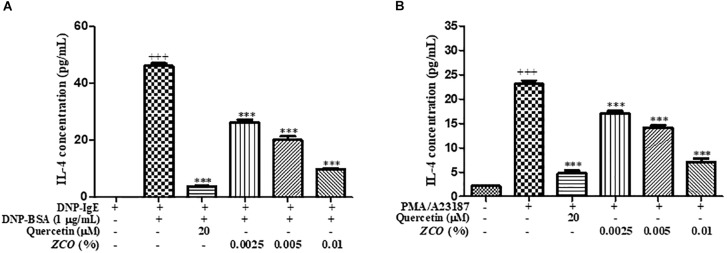
Effect of *ZCO* on IL-4 production in RBL-2H3 cells. **(A)** RBL-2H3 cells were sensitized with IgE overnight at 37°C, followed by pretreatment with *ZCO* (0.0025, 0.005, and 0.01%) or quercetin (20 μM) for 2 h before stimulation with 1 μg/mL DNP-BSA for 16 h. The supernatants were analyzed for IL-4 production. Experiments were conducted in quadruplicate and expressed as mean ± SEM (^∗∗∗^*P* < 0.001 versus with DNP-BSA group). **(B)** RBL-2H3 cells incubated into 48-well plate overnight were pretreated with *ZCO* or quercetin for 2 h prior to stimulation with 30 ng/mL of PMA plus 350 ng/mL of A23187 for 16 h. The supernatants were collected and IL-4 cytokine was tested by using ELISA kit (^∗∗∗^*P* < 0.001 versus with PMA/A23187 group). ^+++^means the significance compared with the mock cells (deal with nothing).

Modulation of inflammatory cytokines from mast cells is one of the key indicators of reduced allergic symptoms, and IL-4 is considered the most important therapeutic step for allergic inflammation diseases (Hoesel and Schmid, 2013). So, we examined whether *ZCO* could regulate a pro-inflammatory cytokine IL-4 in RBL-2H3 cells. The inhibitory effects of *ZCO* on IL-4 release were measured in sensitized RBL-2H3 cells stimulated by DNP-BSA or PMA/A23187. *ZCO* inhibited IL-4 secretion from DNP-BSA-stimulated RBL-2H3 cells with an IC_50_ value of 0.0034%, or PMA/A23187-stimulated RBL-2H3 cells with an IC_50_ value of 0.0059% (Figure 3), suggesting that *ZCO* can modulate mast cell-mediated allergic inflammatory responses.

### Effect of *ZCO* on the Production of Inflammatory Mediators in LPS-Activated RAW264.7 Cells

**FIGURE 4:**
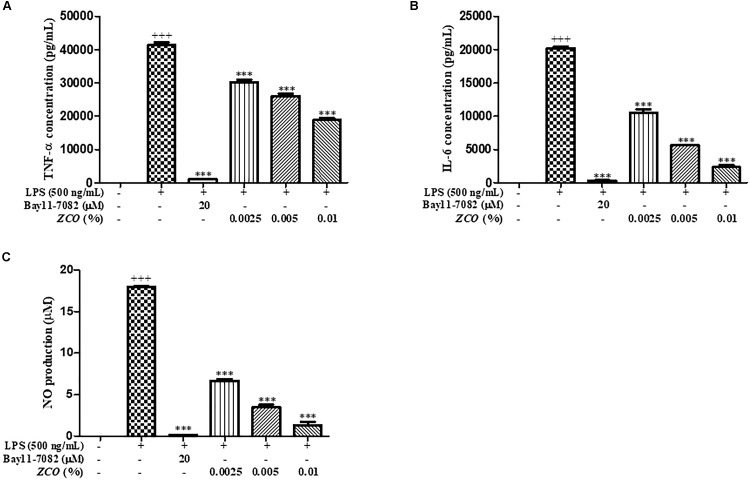
Effect of *ZCO* on the production of inflammatory mediators in LPS-activated RAW264.7 cells. RAW264.7 cells seeded into 48-well plate were pretreated with *ZCO* (0.0025, 0.005, and 0.01%) or Bay11-7082 (20 μM) for 2 h prior to addition of LPS (500 ng/mL) for 24 h. TNF-α **(A)** and IL-6 **(B)** in the supernatants was measured by ELISA and NO production **(C)** in the supernatants was detected by Griess reagent. Experiments were conducted in quadruplicate and expressed as mean ± SEM (^∗∗∗^*P* < 0.001 versus with LPS group). ^+++^means the significance compared with the mock cells (deal with nothing).

To investigate the anti-inflammatory effects of *ZCO*, their ability to modulate NO synthesis, TNF-α and IL-6 cytokines were measured in LPS-stimulated RAW264.7 cells. The results demonstrated that *ZCO* exhibited marked activity against TNF-α release in a dose-dependent manner, with an IC_50_ value of 0.0068% (Figure 4A). In addition, pre-treatment with *ZCO* significantly inhibited IL-6 secretion, with an IC_50_ value of 0.0023%, which were significantly different from the control group. The suppressive effects of 0.01% of *ZCO* approached the effect of the reference compound Bay11-7082 at 20 μM (Figure 4B). NO production was also significantly decreased by *ZCO*, with an IC_50_ value below 0.0025%, which implied anti-inflammatory activity in LPS-induced RAW264.7 macrophage cells (Figure 4C).

### Effect of *ZCO* on the Expression of COX-2 and iNOS in LPS-Activated RAW264.7 Cells

**FIGURE 5:**
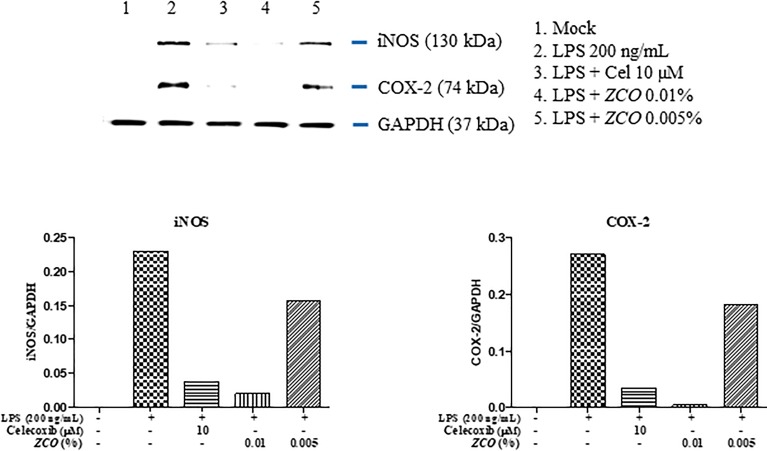
Effect of *ZCO* on the expression of COX-2 and iNOS in LPS-activated RAW264.7 cells. RAW264.7 cells cultured into 6-well plate were pretreated with *ZCO* (0.01 and 0.005%) or celecoxib (10 μM) for 2 h, and then LPS (200 ng/mL) was added to cells for 16 h. The protein levels of iNOS and COX-2 were detected by Western blot analysis. Celecoxib was used as a control for the COX-2 inhibitor, β-actin was used as the control protein.

Pro-inflammatory enzymes, such as iNOS and COX-2, cause inflammation. Thus, we also examined whether *ZCO* could affect iNOS and COX-2 protein expressions by Western blot analysis. RAW264.7 cells were pretreated with *ZCO* (0.01 and 0.005%) or celecoxib (10 μM) for 2 h prior to addition of LPS (200 ng/mL). Stimulation of the RAW264.7 cells with LPS resulted in accumulation of iNOS and COX-2 proteins, as determined by Western blot analysis. *ZCO* significantly reduced the levels of iNOS protein. Also, *ZCO* decreased the COX-2 protein expression in LPS-activated RAW264.7 cells, especially the 0.01% of *ZCO* showed the remarkable effect on the inhibition of COX-2 and iNOS expression (Figure 5).

### Effect of *ZCO* on PMA-Induced NF-κB Activation in 293T Cells

**FIGURE 6:**
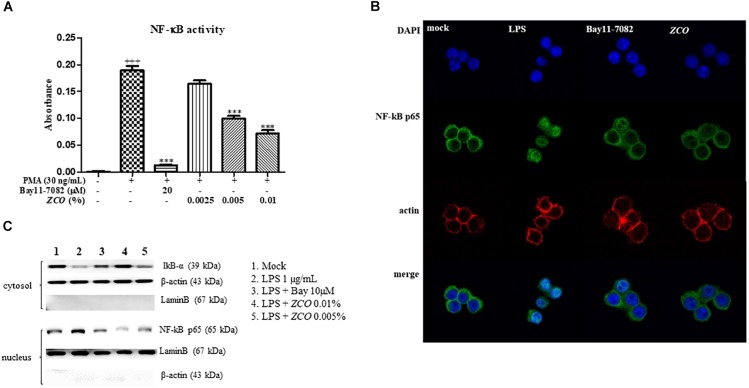
Effect of *ZCO* on NF-κB transcription and translocation. **(A)** Effect of *ZCO* on PMA-induced NF-κB activation in 293T cells. 293T cells seeded into poly-D-lysine hydrobromide coated 96-well plate were transfected with a reporter plasmid, pNF-κB-SEAP for 4 h. Cells were treated with *ZCO* (0.0025, 0.005, and 0.01%) or Bay11-7082 (20 μM) for 2 h prior to stimulation with 3 ng/mL of PMA for 24 h. The supernatants were collected for NF-κB activity assay. Experiments were conducted in quadruplicate and expressed as mean ± SEM (^∗∗∗^*P* < 0.001 versus with PMA group). **(B)** Effect of *ZCO* on NF-κB translocation into nucleus. RAW264.7 cells in 8-well glass chamber plate were pre-incubated with *ZCO* (0.01%) or Bay11-7082 (20 μM) for 2 h prior to addition of LPS (1 μg/mL). The cells were immunostained with a polyclonal anti- NF-κB p65 antibody and Alexa Fluor 488-conjugated 2nd antibody. Bay 11-7082 was used as a control for the NF-κB inhibitor. **(C)** Cytosolic and nuclear fractions were isolated from RAW264.7 cells. The protein level of NF-κB p65 in the nuclear fractions and IkB-α in the cytosolic fractions were detected by Western blot analysis. ^+++^means the significance compared with the mock cells (deal with nothing).

NF-κB is an important factor in the regulation of immune responses in allergic diseases, and has been considered a prototypical pro-inflammatory signaling pathway (Kim et al., 2017). The effect of *ZCO* on NF-κB activation was tested in 293T cells. PMA increased NF-κB transcription, which was decreased by *ZCO* in a dose-dependent manner, with an IC_50_ value of 0.0065%. *ZCO* at the concentration of 0.01% showed the most significant reduction on the activation of NF-κB transcription (Figure 6A).

### Effect of *ZCO* on NF-κB Translocation in LPS-Activated RAW264.7 Cells

RAW264.7 cells were pretreated with *ZCO* (0.01%) or Bay11-7082 (20 μM) for 2 h prior to the stimulation of LPS (1 μg/mL). The effect of *ZCO* on LPS-induced NF-κB pathway was tested. LPS caused the translocation of NF-κB from the cytosol to the nucleus, which was inhibited by *ZCO* (Figure 6B). We also confirmed NF-κB translocation by Western blot analysis. Cytosolic and nuclear fractions were isolated from RAW264.7 cells. Treatment with *ZCO* decreased the level of NF-κB p65 proteins in the nuclear fractions and elevated the level of IkB-α in the cytosolic fractions (Figure 6C). These results indicated that *ZCO* exerted an anti-allergic inflammation effect through the inhibition of NF-κB translocation by the induction of LPS.

### Effect of *ZCO* on MAPKs in LPS-Activated RAW264.7 Cells

**FIGURE 7:**
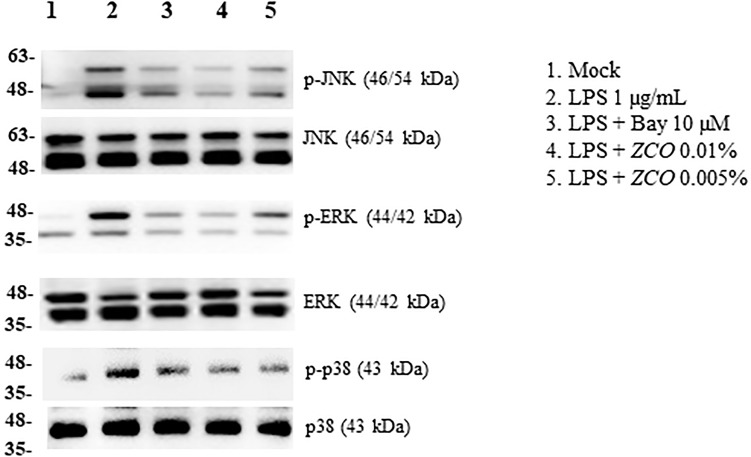
Effect of *ZCO* on MAPKs in LPS-activated RAW264.7 cells. RAW264.7 cells cultured into 6-well plate were pretreated with *ZCO* (0.01 and 0.005%) or Bay11-7082 (10 μM) for 4 h, and then stimulated with LPS (1 μg/mL) for 30 min. Cell lysates (50 μg) were used for Western blotting of the target proteins. Phosphorylated MAPKs were detected by using anti-phospho-JNK, anti-phospho-ERK or anti-phospho-p38 antibody. The membrane was stripped and reassessed with the antibodies targeting non-phosphorylated MAPKs.

Apart from NF-κB, MAPKs are also upstream modulators of inflammatory mediators and cytokines responses. In particular, JNK, ERK, and P38 play important roles in the activation of NF- κB (Huang et al., 2012; Yu et al., 2018). To further investigate whether *ZCO* exerted the inhibitory effects on inflammation through regulating MAPK pathway, we examined the MAPK phosphorylation by Western blot analysis. RAW264.7 cells were pretreated with *ZCO* for 4 h, and then stimulated with LPS (1 μg/mL) for 30 min. Phosphorylation levels of JNK, ERK, and p38 were increased in cells treated with LPS alone, which were remarkably inhibited by *ZCO* treatment. Non-phosphorylated JNK, ERK, and p38 have no changes in cells treated with LPS or LPS and *ZCO* (Figure 7). These results indicated that *ZCO* exerted an anti-allergic inflammation effect via the inhibition of MAPK pathway in LPS-stimulated RAW264.7 cells.

### Effect of *ZCO* on Atopic Dermatitis in Mice

**FIGURE 8:**
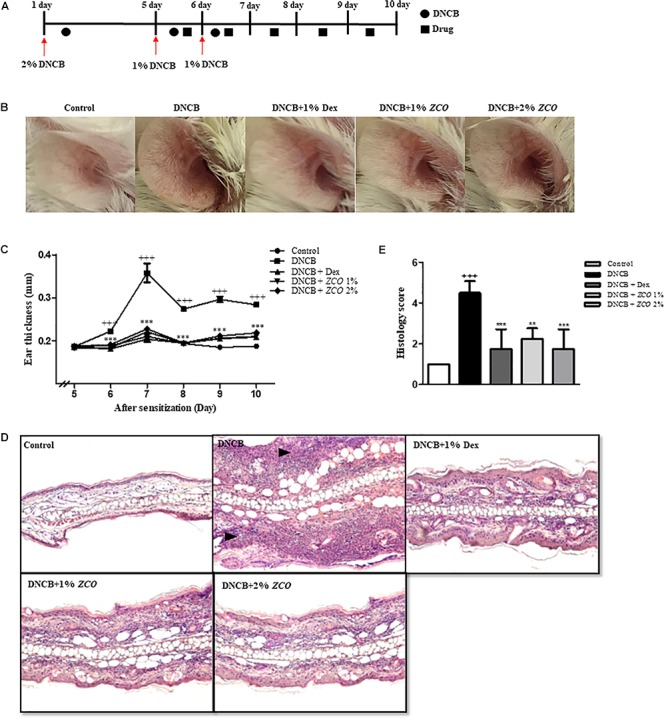
Effect of *ZCO* on Atopic Dermatitis in Mice. **(A)** Experimental design for the induction of AD. 5 days after the first boost with 2% DNCB (150 μL/mouse) to the abdomen, 1% DNCB was applied to both sides of the ears on day 6 and 7. Drug were pre-applied to the ear for 30 min. After 3 h, repeated one time. From day 8, drugs were applied only. **(B)** Representative photographed example of the effects of *ZCO* on DNCB-induced AD in the ear. **(C)** Ear thickness was daily measured with a digimatic micrometer. Data are presented as mean ± SEM (^∗∗∗^*P* < 0.001 versus with DNCB group). **(D)** Histological analysis of AD mice. Representative photomicrographs of ear sections were stained with H&E staining (original magnification: × 100). **(E)** Histological changes (acanthosis, thickening of the subepidermal layer, cellular infiltration) were evaluated semiquantitatively on a grade of 1–5 (1 = no change from negative control group; 5 = maximum change). ^∗∗^means that *P* < 0.01 versus with DNCB group.

We determined the effect of *ZCO* on AD-like skin lesions in mice. DNCB treatment elicited severe ear swelling and AD-like skin lesions such as large ulcers and hyperkeratosis, which were as an index of skin inflammation, and this swelling peaked at day 7 after sensitization. The positive control dexamethasone remarkably decreased the ear thickness, and showed no AD-like skin lesions. In addition, *ZCO* treatment inhibited DNCB-induced ear swelling and AD-like skin lesions comparable to dexamethasone (Figures 8B,C).

A microscopic analysis of the ear by H&E staining showed many alternations. Compared with the DNCB-induced AD-like skin lesions, *ZCO* treatment significantly decreased the DNCB-induced epidermal and dermal thickness and infiltration of inflammatory cells, which was comparable to the positive control dexamethasone (Figures 8D,E).

## Discussion

In the present study, we had screened 15 essential oils from natural plants on anti-allergic inflammatory activities, and the essential oil from *Zanthoxylum coreanum Nakai* (*ZCO*) showed the highest inhibitory activity on the mast cell degranulation. We identified 37 constituents from *ZCO* (Table 1) and β-Ocimene was predominant component (24.48%) followed by (-)-α-pinene (16.56%), 4-carvomenthenol (11.61%), sabinene (10.81%), linalool (10.09%), o-cymene (3.56%), β-phellandrene (3.15%), limonene (2.63%), and α-terpineol (1.74%). Some studies have demonstrated that glycoproteins from *Zanthoxylum piperitum* DC fruits or leaves have the anti-allergic or anti-inflammation effect (Lee and Lim, 2009a,b, 2010; Park et al., 2009). However, there are few researches about the pharmacologic activities of *Z. coreanum.*

IgE-mediated allergic reactions through the FcεRI receptor is known to be the main mechanism of mast cell activation (Krystel-Whittemore et al., 2015). In this study, we investigated whether *ZCO* inhibits mast cell degranulation using RBL-2H3 cells. First, we found *ZCO* profoundly affected anti-DNP IgE-induced local allergic reaction and PMA plus A23187-induced systemic allergic reaction. Degradation of granules in mast cells leads to the release of the enzyme β-hexosaminidase along with histamine. Therefore, β-hexosaminidase is often used as a marker to measure the efficacy of new drugs in preventing mast cell activation and degranulation (Huang et al., 2014; Zeng et al., 2016). Using this assay, we confirmed that *ZCO* significantly inhibited anti-DNP IgE-mediated and PMA/A23187-stimulated β-hexosaminidase release (Figure 2). Mediators such as cytokines (IL-4, TNF-α, etc.) from the cells are involved in the late phase reaction of allergy. Also, IL-4 could modulate the inflammatory response owing to its ability to affect adhesion molecule expression and cytokine production in endothelial cells (Brown and Hural, 1997; Do et al., 2017). Our results exhibited dose-dependent effects of *ZCO* against both anti-DNP IgE-mediated and PMA/A23187-stimulated IL-4 production (Figure 3). These results indicate that *ZCO* may be a promising new anti-allergic inflammatory agent.

Macrophage cells are the key immune cells in the initial period of inflammation. After macrophages are activated by LPS, a series of inflammatory mediators will be released (Jeong et al., 2013; Hu et al., 2014). TNF-α and IL-6 cytokines are key pro-inflammatory cytokines. Therefore, LPS induced macrophages have usually been used for assessing the anti-inflammatory effects of various agents. In this paper, the anti-inflammatory activities of the *ZCO* by reducing inflammatory factors (NO, TNF-α, and IL-6) production in LPS-activated murine macrophages inflammation were studied, and *ZCO* showed the significant reduction on the TNF-α and IL-6 production, respectively, with an IC_50_ value of 0.0068 and 0.0023% (Figures 4A,B). NO is an endogenous free radical which is low in the human body. Inhibition of NO production is usually used as an important treatment for inflammation-related diseases (Zhang et al., 2017). LPS-induced RAW264.7 macrophages could release large amounts of NO, and iNOS is the main enzyme to catalyze NO production in acute and chronic inflammation (Chen et al., 2017). COX-2 is an inducible isoform of cyclooxygenase which mainly exerts its important role in the inflammation (Yi et al., 2013). We found that treatment with *ZCO* dramatically inhibited LPS-induced overproduction of NO in RAW264.7 macrophages (Figure 4C). Consistently, the expression of the iNOS and COX-2 protein were remarkably decreased by *ZCO*, as determined by Western blot analysis (Figure 5).

NF-κB and MAPK pathways are two major mechanisms which regulate LPS-induced inflammatory cytokines production. Activation of NF-κB by the phosphorylation and degradation of IκB-α causes the translocation of NF-κB p65 to the nucleus. This, in turn, activates the transcription of specific target genes such as TNF-α and IL-6 (Liu and Malik, 2006; Hong et al., 2010; Yi et al., 2013). Also, NF-κB induces both iNOS and COX-2 (Park et al., 2004). Therefore, inhibition of NF-κB activity is an important target for anti-inflammation treatment. In this study, *ZCO* significantly inhibited PMA-induced NF-κB activity in 293T cells (Figure 6A). In addition, LPS-induced translocation of NF-κB p65 to the nucleus was significantly reduced by *ZCO* in RAW264.7 cells (Figures 6B,C). This means that NF-κB pathway is involved in the anti-inflammatory effects of *ZCO*. MAPKs phosphorylation are also crucial events in the allergic inflammation response. The maximal MAPK (JNK, ERK, p38) expression is known to occur 10 ∼ 30 min after LPS stimulation (Reddy and Reddanna, 2009; Song et al., 2018). RAW264.7 cells were pretreated with *ZCO* for 4 h, and then stimulated with LPS for 30 min. We found that LPS-induced overexpression of phosphorylated JNK, ERK, and p38 levels was reduced by *ZCO* treatment (Figure 7). These results suggest that *ZCO* exerts anti-allergic inflammatory effects by inhibition of NF-κB activation and MAPK phosphorylation.

Atopic dermatitis (AD) is a chronic allergic skin inflammation disease associated with a combination of intense pruritus, eczematous skin disorder, scratching, and cutaneous sensitization with allergens (Choi et al., 2013). Our further investigation also researched the effects *ZCO* of on DNCB-induced AD-like symptoms in mice model, and found that *ZCO* treatment inhibited DNCB-induced ear swelling and AD-like skin lesions (Figure 8).

## Conclusion

The present study reveals that the *ZCO* inhibits the degranulation and IL-4 production both in IgE/Ag and PMA/A23187-activated mast cell. In addition, *ZCO* is an effective inhibitor of LPS-induced pro-inflammatory mediators in RAW264.7 macrophages through suppressing the NF-κB and MAPK signal pathways. *In vivo*, *ZCO* treatment inhibited DNCB-induced ear swelling and AD-like skin lesions. These findings suggest that *ZCO* may serve as a potential therapeutic candidate for the treatment of anti-allergic inflammatory disease by the inhibition of NF-κB activity and MAPKs phosphorylation. Further studies are needed to characterize some pharmacologic activities of the 37 Chemical compositions validated from *ZCO*.

## Author Contributions

RG carried out major experiments and drafted the manuscript. JP and SJ supported with the study design and reviewed the protocol. JHA and JHP did the histological analysis. JY and SL carried out the essential oil extraction. YK and MP conceived the project, provided reagents and conceptual design, and wrote the manuscript.

## Conflict of Interest Statement

The authors declare that the research was conducted in the absence of any commercial or financial relationships that could be construed as a potential conflict of interest.
